# Interpretation of omics data analyses

**DOI:** 10.1038/s10038-020-0763-5

**Published:** 2020-05-08

**Authors:** Ryo Yamada, Daigo Okada, Juan Wang, Tapati Basak, Satoshi Koyama

**Affiliations:** Unit of Statistical Genetics, Center for Genomic Medicine, Graduate School of Medicine, Kyoto University, Nanbusogo-Kenkyu-To-1, 5F, 53 Syogoin-Kawaramachi, Sakyo-ku, Kyoto 606-8507 Japan

**Keywords:** Data mining, Transcriptomics

## Abstract

Omics studies attempt to extract meaningful messages from large-scale and high-dimensional data sets by treating the data sets as a whole. The concept of treating data sets as a whole is important in every step of the data-handling procedures: the pre-processing step of data records, the step of statistical analyses and machine learning, translation of the outputs into human natural perceptions, and acceptance of the messages with uncertainty. In the pre-processing, the method by which to control the data quality and batch effects are discussed. For the main analyses, the approaches are divided into two types and their basic concepts are discussed. The first type is the evaluation of many items individually, followed by interpretation of individual items in the context of multiple testing and combination. The second type is the extraction of fewer important aspects from the whole data records. The outputs of the main analyses are translated into natural languages with techniques, such as annotation and ontology. The other technique for making the outputs perceptible is visualization. At the end of this review, one of the most important issues in the interpretation of omics data analyses is discussed. Omics studies have a large amount of information in their data sets, and every approach reveals only a very restricted aspect of the whole data sets. The understandable messages from these studies have unavoidable uncertainty.

## Omics studies

In omics studies, a particular type of molecule in samples is measured in terms of character and quantity as a whole, and the patterns and/or relation to the sample attributes are investigated. Genomic studies measure DNA molecules, whereas epigenomic, transcriptomic, proteomic, and metabolomic studies measure the chemical states of DNA and its binding proteins, RNA, proteins, and metabolites, respectively. The concept of –omics, or collective measurement, is applied not to molecules, but rather to various measurable targets, such as a set of traits (phenome), states of brain neural networks (connectome), and bacterial florae (metagenome) [1].

In omics studies each experimental instance generates a huge amount of information simultaneously. For example, a next-generation sequencing (NGS) experiment produces billions of short reads for a genomic, epigenomic, or transcriptomic study [2]. Another example is gas chromatography mass spectrometry for a metabolomic study that produces a spectrum that contains all of the information of various metabolites [3].

## Principles of data-handling: good laboratory practice for omics studies

For small-scale manual experiments, (i) defining, storing, and archiving the raw data; (ii) transparent descriptions of data processing steps; (iii) software validation; and (iv) ensuring complete reproducibility of the final results with respect to the raw data are recommended as good laboratory practice [4]. In addition, unlike small-scale experiments, omics requires (v) checking the distribution of all data values and of their quality measures, as well as the consideration of batch effects [5], so that the records that could be considered to have poorer quality are included in the analyses with probabilistic interpretation. In the following sections, this additional principle will be described for the various steps processing steps.

## Inability to redo omics experiments and intra-experimental and inter-experimental quality variations: batch effect and filtering threshold

### Inter-experimental quality variation

**Fig. 1:**
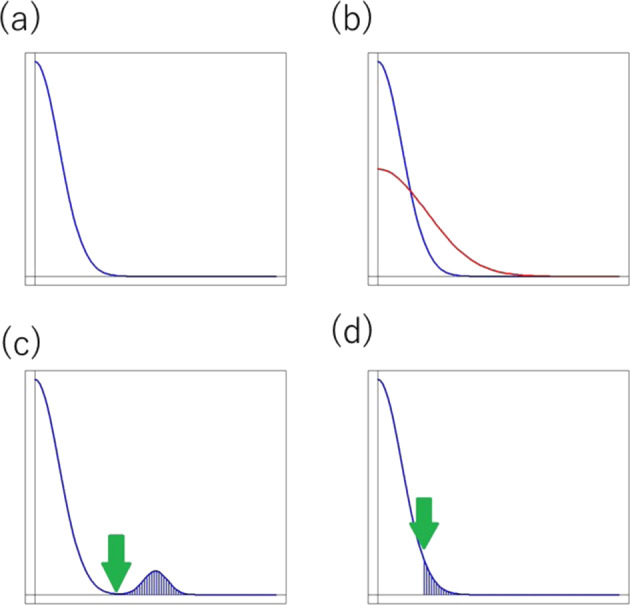
Distribution of quality. **a** Intra-experimental quality heterogeneity. When many data records are produced by a single experiment, the quality of these records can vary. The majority of the records are good, but a tail of poorer quality is generated due to multiple factors. **b** Inter-experimental quality heterogeneity is shown as the difference of quality distributions. **c** When the quality of a small fraction of experiments is apparently poorer than the majority, a particular cause is strongly suspected, which gives good reason to remove the fraction of the experiments by setting a threshold to discriminate these experiments. **d** When the quality distribution is smooth, the selection of a threshold value can be arbitrary

Experiments are not always perfect when conducted manually on a small scale or when performed with expensive highly automated high-throughput machines, which are both true cases for omics experiments. The main difference between these two settings is that manual experiments can be re-performed, but omics experiments cannot be repeated even if the quality of a small fraction of the outputs is unsatisfactory, because the selected fraction cannot be repeated separately. One omics experimental procedure corresponds to a large number of single experiments conducted simultaneously and the quality among the experiments can vary (Fig. 1a). This is referred to as intra-experimental quality heterogeneity. In addition, a set of data records from one omics experimental procedure is affected by factors shared by all of the records, and another set of data records from another omics procedure are affected differently (Fig. 1b). This is referred to as inter-experimental heterogeneity and the procedure-dependent batch effect.

For example, in the case of an NGS, the quality among the reads always varies, i.e., intra-experimental quality heterogeneity exists (Fig. 1a). When two NGS are run for two DNA samples, the first set of reads of the NGS may tend to be better than the second set of reads, for example, because the DNA sample conditions are different. This is referred to as inter-experimental heterogeneity (Fig. 1b).

### Intra-experimental quality variation

There is intra-experimental heterogeneity in data quality in addition to the inter-experimental heterogeneity. Many experiments are performed on a single occasion, and the quality of the experiments can vary. Some data records are filtered out with a cut-off value of a quality measure (Fig. 1c, d; the arrows indicate cut-off values and the shadowed reads will be removed). This procedure may decrease the concern that the poor quality data should produce wrong results. However, the concern cannot be eliminated completely, because some data records that are filtered-in still have some ambiguity. In the omics settings, the output based on the higher quality should be considered to be closer to the truth and the output based on the poorer quality should be considered to be more likely to be farther from the truth. Since the stricter quality requirement reduces the number of usable data records and because fewer records makes the output more likely to be farther from the truth, the filtering cutoff should set so as to balance its effects on the output regarding reliability. Regardless of the filtering threshold, there exists quality heterogeneity among data records to be used for analyses and the quality of individual records can be used in interpreting the output, depending on whether poorer records are instrumental in obtaining the output. This interpretation cannot be performed dichotomously, but rather in a stochastic manner [6].

## Quality control of data: absolute quality and relative quality

The brief discussion on the inter- and intra-heterogeneity of data quality in the previous section is based on the belief that the quality of data records is absolutely meaningful. In this section, two issues are discussed regarding quality measures: [1] absolute quality vs. relative quality and [2] whether noisiness of quality can be the target of study.

### Absolute and relative quality

**Fig. 2:**
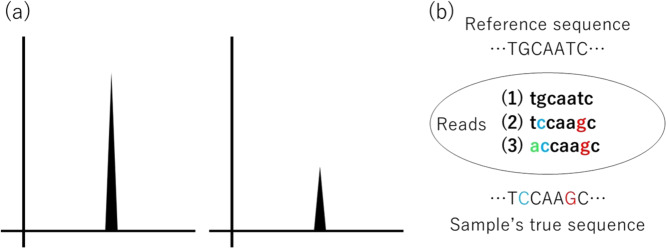
Absolute quality and relative quality. **a** Example of absolute quality. Intensity of physical measure, for example, fluorescence intensity, may indicate the quality of observation. **b** Example of relative quality. The registered reference sequence and the true sequence of a sample are shown at the top and bottom, respectively, by uppercase letters. The different bases are indicated by colors. Three experimental reads are shown in the middle of the figure by lowercase letters. The first read is of the best quality when compared with the reference sequence, but may be of poor quality for this particular sample. The quality of the second read is best for this particular sample, but when the true sequence of a sample is unknown, the quality cannot be determined. The third sequence only has one base mismatch with respect to the true sample sequence but has three mismatches with respect to the reference sequence. The quality of this read is somewhat poor for this particular sample and might be too poor compared with the reference sequence

The majority of experiments measure physical signals, such as light-signal strength, weight, length, or location in space and time (Fig. 2a). Stronger signals or closer measurements to precise values indicate better quality. Let us refer to this type of quality as absolute quality.

The quality of data records can be measured as the fitness to a representative or standard in the life science field. For example, short reads are data records of NGS, and the reference genome sequence is the representative (Fig. 2b). Although genome sequences of individuals have a great deal in common with the reference sequence, some sequences are definitely different from the reference because of genetic heterogeneity. Therefore, short reads that are perfect from an absolute quality standpoint can have a non-perfect matching measure when compared with the reference. The number of unmatched bases can be used to quantify the goodness of reads, and the mismatches can be due to poor quality in the sense of absolute quality, but may be due to biological heterogeneity. The latter can be referred to as the relative quality. These relative quality measures should be handled carefully in order to optimize the study objectives. In the mapping step of NGS, the choice of mapping algorithm and the setting of parameter values should be optimized [6].

### Noisiness can be the target of biological studies

**Fig. 3:**
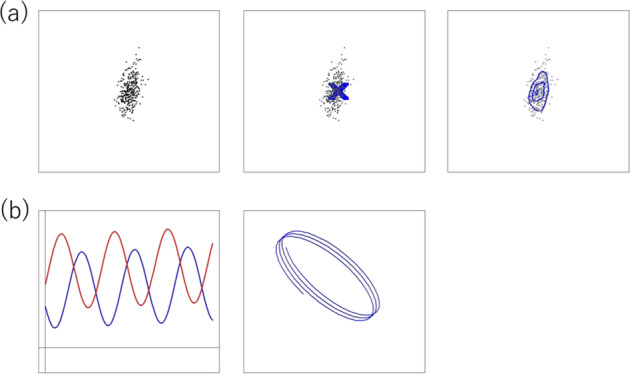
Noisiness. **a** Assume the observation on the left panel. When this observation is obtained as multiple signals from one point, as indicated in the middle panel, the deviation of dots from the cross should be interpreted as random noise. When the observation is obtained as multiple samples from a population with heterogeneity in terms of this measurement, the variation of dots is the most important information. The population’s heterogeneity is represented by contour lines in the top right panel. **b** Example of source of noisiness. The bottom left panel shows the chronological periodic change of the expression level of two genes, X and Y. The right bottom panel is a two-dimensional display of the positions of (X, Y). When a particular cell type is studied and many cells of this type of cycle are evaluated for X and Y, individually (single-cell expression analysis), their coplot of (X, Y) will appear circular. This is not noise, but rather an important feature of this type of cell. However, if these value sets of X and Y are observed without chronological order, this variation can be considered as noise without meaningful explanation

The sequence heterogeneity of DNA can be the target of a biological study. The difference of expression profiles between cancer and normal cells is the target of a biological study [7]. The expression profiles of cancer cells are not identical, but rather have a distribution, and the cells from a normal region have another distribution. We may find a small number of cells that are significantly deviated from the two distributions. This deviation can be due to an experimental outlier in terms of absolute quality. However, the deviation can be due to the biological truth. A small fraction of cells is deviated from the two reference cell groups, i.e., exhibit heterogeneity in the sense of relative quality. Actually, cellular expression profiles are noisy or heterogeneous because expression profiles change dynamically along with their chronological, metabolic, and other kinds of conditions. This heterogeneity is the principal motivation of single-cell studies [8] (Fig. 3)

.

### Intensity of interests in the heterogeneity varies among studies

**Fig. 4:**
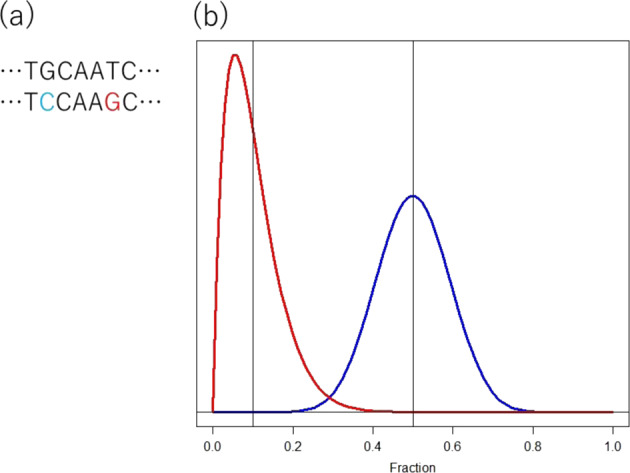
Intensity of interest in heterogeneity. The two sequences stand for an SNV at the second bases, G/C. When genomic DNA of an individual who is heterozygous at this SNV is sequenced for genetic variants with NGS, a fraction of short reads with C at the SNV site vary around 0.5, as indicated by the black line. When the depth is lower, the distribution is fatter. When the number of reads with C is 13 out of 30, it is reasonable to refer to this individual as heterogeneous at this SNV. However, when the number of reads with C is 3 out of 30, it is safe not to believe that this individual is heterozygous. In this case, the researchers are interested in the fraction around 0.5 and 3 out of 30 is considered as a noise. When a cancer researcher who is interested in the fraction of cancer cells that is heterozygous at a site, the cancer cells are sequenced and the fraction is determined to be 10%. The researcher does not ignore this finding because a small fraction of cancer cells has the C allele, and the expected distribution of this fraction may take the distribution indicated by the blue line

Let us continue to use the single-cell expression profiles from two regions. When there is a gross difference between two cell groups in two regions, e.g., cancer and normal regions, the small fraction of cells that do not belong to either of the two cell groups may be out of the interest of the researchers. The researchers may ignore the outlier-looking cells. In contrast, the same experiment can be designed by researchers who are interested in the expression profile dynamics of these cells. The researchers would carefully check the absolute quality of the small fraction of cells. Subsequently, the approaches to the raw data of the two research groups should be different [9]. Figure 4 describes the example how variation of interests of studies affects on the interpretation of experimental outputs.

## Main analyses

Once the pre-processing step has been completed, the processed data should be handed to the main analysis step. Since every omics study layer is mutually different in terms of data structure and objectives, the analysis methods are different. In this review, these omic-layer specific issues are beyond the scope of the present study and the points that are shared by all of the layers are discussed with respect to the following two aspects: [1] the method by which to handle individual items and [2] classification from statistics and learning attitudes.

### Evaluation of Individual items: one by one vs. collapse of the whole

#### Evaluation of individual items one by one

##### Individual evaluation

In omics studies, long sequential molecules, many genes, genetic variants, molecules, biomarkers, cells, and individuals are treated. One way to manage these items is to investigate each element one by one and to obtain many pieces of outputs. This approach includes GWAS in which many single nucleotide variants (SNVs) are studied one by one and transcriptomic RNAseq analysis in which all coding genes are quantified. When these individual items are statistically tested for independence of a particular variable, many *p* values are generated. This set of *p* values should be interpreted as a whole, and this issue is called multiple testing correction, as discussed in the next sub-section. Multiple testing correction segregates the items into two subsets: one subset is positive, and the other subset is negative. However, such segregation of items is insufficient, and the combinatorial effects of multiple items can be the target of studies. Since the number of items in omics studies is very large as compared with the number of variables in conventional multivariate analyses, a statistical approach to combinatorial effects in omics studies is much more challenging than conventional combinatorial problems and is an active sub-field of research [10].

##### Multiple testing correction

When a list of *p* values is generated with the item-wise testing approach, the *p* values cannot be interpreted in the same manner, where only one statistical test is performed and only one *p* value is obtained. This is because 10,000 SNVs, i.e., 1% of a million SNVs in GWAS, will exhibit *p* values that are smaller than 0.01, even when all SNVs are independent of the phenotype of GWAS [11]. The rarity of the smallest *p* value among the million *p* values should be interpreted not in the uniform distribution from 0 to 1 but rather in the distribution of smallest values among the million random values that follow the uniform distribution from 0 to 1 [12]. Based on this rarity calculation, the minimum *p* value should not be considered rare when the rarity is 10^(−6)^, but 10^(−8)^ can be considered to be sufficiently rare. This method of rarity correction is referred to as family-wise error rate (FWER) correction [13], which is suitable for GWAS, because the vast majority of SNVs should not be associated with a particular phenotype.

**Fig. 5:**
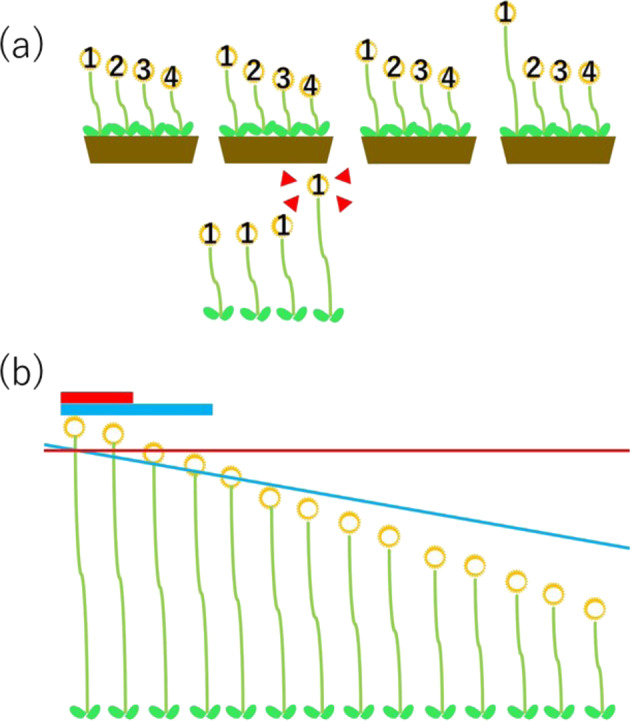
Multiple testing correction. **a** Family-wise error rate (FWER). When multiple items (flowers) are observed, their values (heights) vary. The tallest flower in each group can be significantly tall, but its height should not be used for judgment. The height should be judged among the tallest flowers from many groups. This method of significance judgment is applied to the interpretation of the rarity of *p* values and is referred to as FWER correction. **b** In (**a**) there are only four flowers in a group. (**b**) has many more flowers. The FWER applies the same cut-off value (red line) to all flowers, and the first and second tallest flowers are judged to be significantly deviated. The false-discovery rate method applies different cut-off values (blue line) depending on the rank of the flowers and the top four flowers are judged to be significantly deviated

In contrast, there are studies in which a considerable number of items could be truly associated with a phenotype, for example, a study to compare the expression level of 20,000 genes between two distinct cells, e.g., cancer cells and normal cells in an organ. In this case, the minimum *p* value from 20,000 tests is interpreted in the same manner with FWER, because before evaluating the minimum *p* value, we do not have any idea as to whether any item is truly associated. Once the minimum *p* value is judged to be truly associated, we have a good reason to believe that two cell groups are different in terms of gene expression profiles. At this point, we believe that a gene with a minimum *p* value is truly positive, and the total number of null hypotheses is not 20,000, but rather 19,999. Therefore, the interpretation of rarity of the second smallest *p* value should not be the same are the smallest p value, and the larger threshold value should be set as the second smallest *p* value to call this value positive. This thinking process makes us apply a less strict threshold to judge the rarity of *p* values depending on their ranks. This approach is realized by the method called false-discovery rate (FDR) correction [13]. Figure 5 illustrates the difference of cut-offs between FWER and FDR correction.

#### Collapse of the whole data set or dimension reduction

##### Dimension reduction

Another approach to the evaluation of many items is to evaluate whole items in order to detect patterns therein. This approach includes clustering samples with expression profiles and principal component analysis (PCA) to identify informative components that consist of a weighted sum of individual items [14]. This approach can also be considered as a dimension reduction approach. Dimension reduction is useful when the observed data set consists of many variables or is distributed in high-dimensional space, but the variables are not scattered randomly in the high-dimensional space, but rather are localized in a subspace. Actually, omics studies are performed based on the belief that biological phenomena are complex and should be described with fewer important principles. Since the subspace is narrower than the whole space, the data set can be described in lower-dimensional space. The approach to collapse the whole data set with a high dimension is the approach to find fewer components to describe the whole data set [15].

##### Linear vs. non-linear methods

When a simple transformation of the whole data set finds a lower-dimensional subspace into which the majority of the whole is mapped, linear dimensional reduction methods, such as PCA and multi-dimensional scaling, are appropriate. When complex transformation is required to find such a subspace, nonlinear dimension reduction methods are necessary. Multiple nonlinear embedding methods have been developed and proposed; for examples, tSNE seemed to get popular in single-cell transcriptome and UMAP was proposed as another one that stresses different features of original high-dimensional distribution [16]. Linear methods are simple and straightforward with few variations and also simply interpreted. In contrast, nonlinear methods are flexible and vary greatly, and output interpretation requires knowledge of their mechanisms. (See the discussion on linear and nonlinear methods for data visualization).

The selection of linear and nonlinear dimension reduction methods is problematic in real data analyses because the whole data structure cannot be “seen” in high-dimensional space. When the whole data set is in two- or three-dimensional space, we may visualize the data and determine whether a method would be most appropriate. However, when the whole data set is in high-dimensional space, the structure cannot be seen, and there is no clue by which to select an appropriate method.

##### Decomposition/non-decomposition into orthogonal/independent components

Dimension reduction provides fewer components by which to describe the whole data set. When the identified components are mutually independent or have no overlap in terms of the aspects to describe the whole, the components are mathematically elegant and easy to interpret. When the axes are mutually independent from an information standpoint, the axes make right angles to each other, i.e., are orthogonal. Therefore, many mathematically sound dimension reduction methods are designed to find orthogonal components.

However, such mathematically elegant components can be difficult to understand in natural language (see Section 6). Another approach to dimension reduction is to select components that are not necessarily independent each other but are easy to understand, such as a list of biological functional subsets of genes. As far as such components describe the whole data set with reasonable adequacy, it can be said that the whole data set is reduced to a space with fewer components [17].

##### Dimension reduction as an intermediate processing

Dimension reduction methods are powerful. However, even after the dimension reduction, the output can be of relatively high dimension or can appear complicated. This happens frequently in omics studies because the dimension of the original data sets is significantly high.

In this case, the output of dimension reduction has to be analyzed further, and the dimension reduction step is considered as intermediate processing.

### Classification of methods from statistics and learning attitudes

There are four ways to say something meaningful using an omics data set. The contents of this section are true for any kind of data analyses and are not specific to omics data analyses. The first approach is a statistical test that rejects the null hypothesis and supports the alternative hypothesis. The second approach is to estimate a meaningful relation or to generate a predictive model. The third approach is to identify a pattern in a data-driven fashion. Finally, the fourth approach is to use a data set as information to update a prior belief to a posterior belief in a Bayesian framework.

#### Statistical test

Scientific studies try to find novel things that we find difficult to believe without supporting evidence. In this case, the probability of observing evidence through statistical tests is too low if we do not believe the novel finding, but believe the hypothesis that denies the finding, i.e., the null hypothesis. The rarity of the evidence when we believe the null hypothesis is quantified as the *p* value [12]. When testing the association between the genotypes of a genetic variant and dichotomous phenotypes, the null hypothesis is that genotypes and phenotypes are mutually independent and the *p* value of its independence test measures the rarity of the table observation if the null hypothesis is true.

#### Statistical estimation and machine learning of a predictive model

An independent test of the above-mentioned genotype-phenotype table can be used to estimate the genotypic risk ratio (GRR), which should be 1 if there is no association between genotypes and phenotypes, but GRR deviates from 1 if the null hypothesis should be rejected [18]. The GRR is the relative risk to develop a particular phenotype of one genotype against other baseline genotype. Although we want to know the true value of the GRR, it appears to be impossible to determine the GRR with a limited amount of information, and its value must be estimated. Sometimes one representative value of the GRR is estimated, which is referred to as the point estimate, and sometimes there is a range within which the true value is believed to exist with residual uncertainty, which is referred to as the interval estimate [19]. In this case, we estimate GRR, but when two variables, X and Y, are both continuous, the deviation from independence between X and Y is measured as the linear regression slope coefficient in the simplest model. Again, this coefficient should be estimated, and its representative point value and its interval might be estimated. Both the GRR and the slope coefficient are quantified effects or effect sizes. When we estimate the effect size of something, we believe that there exists a sizable effect, rather than no effect (null hypothesis). The estimated effect is sufficiently small, and the estimated effect size can be compatible with the null hypothesis. Although these GRRs and the linear regression coefficient are simple examples of estimation based on a data set, they are based on an assumed model and enable us to predict the value or probability of new samples that lack the observation of part of the variables in the model. In this sense, they are simple cases of supervised machine learning [20, 21].

#### Descriptive statistics and unsupervised learning to identify patterns

Supervised learning in the previous section is a machine learning task to generate a particular model to describe the relation of input and output with training samples, and the generated model should work to predict output for new input. As stated here, this requires training samples that have both input and output that should be the answer of the prediction model. In contrast, in some contexts, multiple variables of multiple samples are observed, and all observed data records have some noise and no “answer” is available. In this situation, the main interest in the data set is to extract particular patterns in the data set itself, or to identify deviation from randomness among the variables and samples. This approach is called unsupervised learning [20, 21].

#### Bayesian approach

In a Bayesian approach framework, an assumption or hypothesis is set beforehand, and the hypothesis is updated with the observed data to produce the belief afterward, which becomes the message of the analysis [21]. Since the Bayesian approach is to use data, the data are used in various contexts. For example, a statistical model is set, and the parameter value is estimated by updating the prior value in a Bayesian manner. As mentioned in 5.1.1.2. FWER correction is applied when the null hypothesis is believed to be true for all tests, and FDR correction is applied when a fraction of the items is believed to be associated. In the context of Bayesian interpretation, FDR correction is based on the prior belief of the presence of true positives as compared with the prior belief of flat null hypothesis, which leads to FWER correction. When risk variants are being looked for in GWAS and when assuming that SNVs in exons of a particular subset of genes are more likely to be truly associated with the target phenotype than SNVs in intergenic regions, you should set different thresholds for *p* values of independence tests from SNVs in the two regions. This approach also uses the assumption and, therefore, is Bayesian. This kind of Bayesian thinking may appear in the discussion sections of papers, rather than the methods or results sections, partly because this type of assumption is difficult to sufficiently quantify precisely to be included in a prior update. The prior knowledge can be included in a more systematical manner by designing the mechanism in a pipeline [22].

## Translation of the outputs into perceptible forms

The raw outputs of statistical and machine learning methods are essentially in the form of numbers, formulae, and symbols. The vast majority of people consider the expressions with numbers, formulae, and symbols to be inappropriate as the methods of interpretation. Intellectual interpretation of inputs is rooted on the physical systems of perception. As humans, we have five senses, two of which (the hearing and visual senses) are mainly used for intellectual communication. Information for the hearing sense takes the form of words and languages and that for the visual sense takes the form of two-dimensional graphics. Therefore, we transform the output numbers, formulae, and symbols into the forms of words/language or two-dimensional graphics.

### Annotation and ontology

Two technical systems have been developed to connect the omics information to natural languages in the field of bioinformatics: annotation [23] and ontology [24]. Annotation translates the location in the genome to the gene name and chemical compounds, and their domains are annotated with specific terms. Multiple genes, for example, share a biological function, which is called with a functional term, and the genes of the function can be grouped as a subset of genes with the functional term. In addition, the system of ontology has the information to connect the gene names and the functional term. The ontology system provides the relations among various technical terms, and, using these terms, we can write a meaningful natural language statement. The terms in annotation and ontology systems are defined and registered in databases. Therefore, we can understand the statements with terms, even if we find unfamiliar terms in the statements, by obtaining information of the terms in the databases.

Annotation and ontology systems are very powerful for interpreting the output of analyses. We should not forget the fact that these systems are not perfect and can mislead us. For example, if an SNV is identified as a risk locus of a particular disease and the variant is annotated to be located in a gene structure, the SNV is usually translated as the gene name, “gene-X”, and we state that “an SNV in gene-X is associated with the risk of the disease”. This statement, while true, can be misleading. The SNV is functionally related a neighboring gene, “gene-Y”. Then, the statement misleads the readers. The association between the SNV and the disease may be due to linkage disequilibrium (LD), and the functional truly related to another SNV in the LD block is associated with the disease, and the true SNV may be annotated as an SNV in “gene-Z”. Again, the statement can mislead the readers. Therefore, interpreters should be aware that translation into natural language with annotation and ontology systems are powerful, but have a limitation. The translated statements should be believed as they are in the limitation, and there can be different statements based on identical analysis outputs.

### Visualization

Data visualization is a computational research field and covers all topics to visualize messages and meanings. Data visualization of data analysis results is a part of this field [25]. In this review, two-dimensional visualization of higher-dimensional omics data is discussed [26].

#### Linear methods

**Fig. 6:**
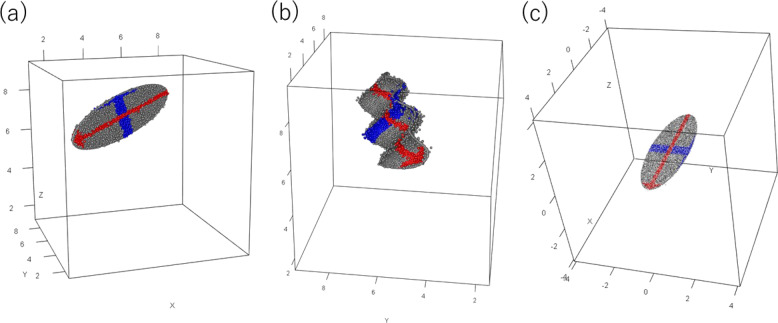
Linear nonlinear transformations. **a** Linear transformation. The data are originally in three-dimensional space. The object of interest is a flat disk with bumps. After linear transformation, the disk is placed in two-dimensional space as shown in (**c**), and the bumps on the disk are perpendicular to the plane of the disk. When the two-dimensional space for the disk is selected for the principal components, the third axis is discarded as the noise axis, and the bumps on the disk are ignored as noise. **b** Nonlinear transformation. The data comprise a wavy disk with bumps on it. A nonlinear method transforms the wavy disk into a flat disk as (**c**), and places the flat disk in two-dimensional space. Again, the bumps on the wavy disk can be discarded as noise

The methods are divided into two types: linear and nonlinear. The representative method in the linear type is PCA and its visualization in the form of a coplot with two principal components. PCA rotates all of the data points in a higher dimension (Fig. 6a) and finds the two most important axes and extracts and displays coordinate values for the two axes (Fig. 6c). In other words, all information out of the two-dimensional plane is discarded. In some cases, more than two principal components are selected for visualization, the selected axes are paired, and coplots of the pairs are displayed. This approach reduces the amount of discarded information but forces the readers to reconstruct the multi-dimensional point clouds from the multiple two-dimensional displays. Since this type of method discards the information in the nonselected axes, why discarding this information is appropriate must be explained. As such, eigenvalues of each component are used. The sum of eigenvalues of the selected components is reasonably large as compared with the total sum of eigenvalues, and the selected components are considered to explain the whole data set adequately. Ignoring other components as random noise is reasonable. Another consideration for this type of method is that only the original points are rotated in the higher-dimensional space, and the mutual distances are maintained [16].

#### Non-linear methods

In contrast, nonlinear methods do not maintain mutual distances (Fig. 6b, c). This means that two points that are mutually closely located in the original higher dimension should be embedded relatively close in two-dimensional space and that two points that are mutually distant in the original space should be embedded relatively distant in two dimensions. Relative closeness is not only maintained, but the distances among the embedded points are also fake. With this allowance of deformation, nonlinear methods have the advantage whereby these methods do not have to discard any directional information, but can use the whole information in the original higher dimension. Since the visual appearance of nonlinear methods is deformed, the viewers have to be able to grasp the information in the deformed view, that is the same where the viewers of deformed art paintings need critical eyes to enjoy such artworks. There are multiple painters of deformed art with their own styles, and multiple nonlinear methods with their own deformation styles can be applied. The viewers of nonlinear embedding should understand the difference in the styles of these methods [12]. The difference of styles can be described as: for example, tSNE realizes the embedding of points by measuring the Euclidean distance among the points in the high-dimensional space and by stretching the visualizing low dimensional space sheet to generate rooms for the points that are located in the very spacious high-dimensional original space. UMAP identifies neighbors in the high-dimensional space to generate a graph and subsequently finds a good placement of the graph in the low dimensional space [27].

#### Graph or network visualization

The above discussion of linear and nonlinear embedding is the visualization of points. A graph consists of points and the edges between the points. Basically, points connected by an edge represent mutually close points. When the relation of points is expressed by a graph and the graph is visualized, both points and edges are displayed in two-dimensional space. Visualized graphs having edges that are shorter and have fewer crossings are easier to view. Many graph visualization methods exist and have their own algorithm or style for visualization. They are somewhat similar to nonlinear two-dimensional embedding, and the difference between graphs and nonlinear two-dimensional embedding is that graphs have additional objects and edges and that graphs may add information on points and edges, for example, the sizes of points and the widths of edges [28].

#### How to view paintings of data analysis outputs

If 10 persons view a deformed painting artwork and record comments on the artwork, the comments will be quite different. It should not happen to the case of visualization of data analysis outputs. The person who shows an output painting is responsible for making the viewers receive a reasonably similar message. As such, the person in charge should guide the viewers as to where to start watching and what should be sensed with descriptive sentences. The guidance should be based on the style of the deformation method.

## Many methods and many tools

There are many data analyses tools available [29]. In addition, multiple combinations of these methods in the form of data processing pipelines have been reported. All of the methods and pipelines return different outputs. These outputs represent aspects of the whole data set, and it is essentially impossible to extract all of the information contained therein. Therefore, all methods and pipelines return their own outputs, which represent their viewpoints, but lack the viewpoints of other methods. In other words, all of the methods and pipelines have advantages and disadvantages. Based on the above-mentioned considerations, the purpose and design of your study should be reconsidered. This will guide your selection and assist interpretation of the results of your analysis.
